# Comparative analysis of ascorbic acid in human milk and infant formula using varied milk delivery systems

**DOI:** 10.1186/1746-4358-3-19

**Published:** 2008-08-11

**Authors:** Jimi Francis, Kristy Rogers, Paul Brewer, Darby Dickton, Ron Pardini

**Affiliations:** 1 Department of Biochemistry, Agricultural Experiment Station, University of Nevada, Reno, NV, USA; 2 Truckee Meadows Community College High School, Reno, NV, USA

## Abstract

**Background:**

The expression of human milk for later use is on the rise. Bottle systems are used to deliver the expressed milk. Research has shown that storage of both human milk and artificial baby milk, or infant formula, leads to a loss of ascorbic acid (commonly called Vitamin C). As milk is removed from the bottle during feeding and replaced by ambient air, it is unknown if loss of ascorbic acid occurs during the course of a feeding. The purpose of this study is to investigate the effect of the milk delivery system on levels of ascorbic acid in human milk and infant formula. The objectives are to 1) determine changes in ascorbic acid concentration during a 20 minute "feed," 2) determine if there is a difference in ascorbic acid concentration between delivery systems, and 3) evaluate if any differences are of clinical importance.

**Methods:**

Commonly available bottles were used for comparison of bottle delivery systems. Mature human milk was standardized to 42 mg/L of ascorbic acid. Infant formula with iron and infant formula with docosahexanoic acid were used for the formula samples. Each sample was analyzed for ascorbic acid concentration at baseline (0), 5, 10, 15, and 20 minutes. Each collection of samples was completed in triplicate. Samples were analyzed for ascorbic acid using normal-phase high performance liquid chromatography.

**Results:**

Ascorbic acid concentration declined in all bottle systems during testing, Differences between the bottle systems were noted. Ascorbic acid concentrations declined to less than 40% of recommended daily intake for infants in 4 of the bottles systems at the 20 minute sampling.

**Conclusion:**

The bottle systems used in this study had measurable decreases in the mean concentration of ascorbic acid. More research is needed to determine if the observed decreases are related to lower plasma ascorbic acid concentration in infants exclusively bottle fed. The decrease of ascorbic acid concentration observed in both human milk and infant formula using varied milk delivery systems may be of clinical importance. For infants who rely solely on bottle feeds there may be increased risk of deficiency. Bottle shape, size, and venting should be considered.

## Background

According to the US Department of Labor, as of March of 2006, 75.3% of women were employed outside the home [1]. Changes in lifestyle and economic activities of lactating mothers have led to an increase in the expression of human milk for later use [2]. Often a bottle system is used to deliver the expressed milk to the infants. While breastfeeding is consistently recommended as the best choice for infant nutrition, the expression, handling, storing, and subsequent provision of human milk to infants introduces the possibility of contamination and nutrient deterioration. Recommendations for human milk handling and storing are typically made to prevent bacterial growth [3,4] rather than maintaining the nutritional quality. Handling and storing of milk introduces the possibility of oxidation and/or degradation of sensitive nutrients such as Vitamin C (ascorbic acid) [5].

The L-enantiomer of ascorbic acid, commonly known as vitamin C, is a nutrient whose deficiency causes the disease scorbuticus (scurvy). This essential nutrient is critical to the health and development of infants as it is necessary for the synthesis of collagen, an important structural component of blood vessels, tendons, ligaments, and bone which develop rapidly during infancy [6]. Ascorbic acid is also a highly effective antioxidant. Even in small amounts it can protect indispensable molecules in the body, such as proteins, lipids, and nucleic acids from damage by free radicals and reactive oxygen species that are generated during normal metabolism and rapid growth. Ascorbic acid is known to spare vitamin E (tocopherols and tocotrienols) [7], which is another essential nutrient required by infants for healthy development.

Ascorbic acid is water soluble, and not stored in the body, making regular intake necessary to prevent scurvy. The ascorbic acid content of food is strongly influenced by handling and storing practices [8]. One reported case of parents boiling milk, which causes a loss of ascorbic acid, provided a dramatic example of scurvy in a toddler [9].

Losses of ascorbic acid during the handling and storage of human milk have been studied. In one report, storage at both refrigerator and freezer temperatures led to a significant decrease in the antioxidant capacity of breast mil [10]. In related reports, total ascorbic acid levels decreased on average by one-third after 24 hours of storage at 4°C, with wide variations between individuals (6 to 76% and 3 to 100%, respectively with N = 11) [11]. Researchers have investigated the effect of storage on quality and oxidative sensitivity of human milk [12]. Further, storage of human milk and infant formula leads to the formation of lipid peroxidation products, such as lipid peroxides, conjugated dienes, and aldehyde breakdown products [13]. These studies indicate that stored human milk and infant formula are susceptible to nutritional degradation and loss of specific nutrients.

It is unknown how much loss of ascorbic acid may occur during the course of a feeding when using a bottle to deliver the milk to an infant. Many women do use bottles to provide expressed human milk to their infants. As milk is removed from a bottle by the suckling infant, ambient air moves into the bottles and through the milk, creating bubbles. It is possible that degradation of ascorbic acid occurs during this process.

The purpose of this study was to investigate the effects of the milk delivery system on levels of ascorbic acid in human milk and infant formula with seven commonly used bottles. The objectives were 1) to determine if there is a decrease in concentration of ascorbic acid during a 20 minute time period, 2) to evaluate any difference in ascorbic acid concentration between bottle delivery systems, and 3) to evaluate, if any, whether differences in ascorbic acid concentrations are of clinical importance to infants' nutrition.

## Methods

### Delivery systems

These seven commonly used bottle systems were compared: the Avent^® ^Natural (AN) bottle, the BornFree™(BF) bottle, the Dr. Brown's Natural Flow^®^(DB) bottle, the Evenflo^® ^Elan (EL), the Gerber^® ^Classic(G), the Playtex^® ^Drop-Ins (PD) bottle, and the Playtex Ventaire^®^(V) bottle were used for the comparisons. The AN, DB, EL, G, and V bottles were made of polycarbonate. The BF bottle was made of glass and the PD had a pre-sterilized disposable plastic liner. A glass screw-top bottle of similar shape and size was used as a Control. The diameter of each bottle was measured in millimeters at the milk-to-air interface with 100 ml of milk (at baseline – 0 minutes) in the bottles using General Calipers. Surface area of the milk-to-air interface was approximated using πr^2 ^based on the diameter measurement. The headspace at baseline was approximated using the total volume minus 100 ml (amount of milk at baseline in each bottle).

### Milk samples

Pooled, mature human milk donated by anonymous volunteers was used for the human milk samples. A sample of the pooled milk was analyzed for ascorbic acid concentration. Stock solution of ascorbic acid was added to the pooled milk to standardize the ascorbic acid concentration to 42 mg/L. Infant formulas, one with high iron and one with docosahexaenoic acid were used for the formula samples. All samples were brought to 25°C in a water bath before sampling. Each bottle was sampled at baseline and at four time points. Except for the Control bottle, the same commercially available electric pumps were used to extract the fluid from the bottles and simulate infant suckling. The pumps were adjusted to deliver the milk at five ml/min. As the milks were delivered through the seven bottles, samples of milk were collected at baseline, and every five minutes for 20 minutes using a sterile pipette. As samples were collected, an aliquot was dispensed into a labelled collection tube. The Control sample of each type of milk was placed in a separate clear glass bottle and capped. Samples were collected from the control bottle of milk via sterile pipette at baseline and every five minutes in the same manner as the pumped milk and an aliquot was placed into labelled collection tubes. Each sample collection was completed in triplicate. The collected fractions of milk were protected from light, flushed with nitrogen, quick frozen in liquid nitrogen, and stored at -80°C until extracted.

The milk fractions were protected from light, thawed to approximately 22°C in a water bath, and then mixed. The samples were extracted as previously described [14]. Briefly, in time intervals of five minutes 300 μl of each milk sample was placed in a micro centrifuge tube. 800 μl of 100 mM DL-Dithiothreitol was added and mechanically mixed by inversion for 30 seconds. The samples were incubated at room temperature for 15 minutes. 300 μl of 0.56% *meta*-phosphoric acid was added and mechanically shaken for 30 seconds and centrifuged at 10°C (10 min, 3000 × g) in amber eppendorf tubes. The mixture was filtered into amber glass vials and stored at -20°C until injection for normal-phase HPLC analysis.

### Analytical methods

Isocratic chromatographic separation was carried out using a mobile phase of Milli-Q water with acetic acid (0.1%, v/v) and methanol in a relative proportion of 95:5 (v/v) using all HPLC grade reagents. 1 ul of filtrate was injected by a WPS-3000 auto-sampler (Dionex) onto a Polar Advantage II (C18 3 um, 4.6 × 150 mm, Dionex) equipped with a guard column (PAII C18, 5 um, 4.3 × 10 mm, Dionex) and chromatographed on the Ultimate 3000 HPLC (Dionex) using the LPG-3000 loading pump in conventional HPLC mode. Ascorbic acid was identified by comparing the retention time of the sample peak to the peak from the ascorbic acid standard at 254 nm.

### Statistical analysis

All values were entered into a Microsoft Excel spreadsheet. The mean of each triplicate sample set was calculated. The mean values were used to calculate the amount of ascorbic acid in milligrams per liter of milk. Standard deviation and percentage of baseline was calculated for each mean value. Analysis of variance was used to determine significant differences in ascorbic acid levels for the time periods being studied and to analyze the association with headspace and milk-to-air interface. Alpha error was set at p < 0.01 for all tests.

## Results

### Delivery systems

Not all the bottles were consistently shaped. Therefore, the headspace volume and the milk-to-air interface area were best approximations. The diameter of the bottles varied from 50 mm to 67 mm as seen in Table 1. The headspace ranged from a high of 205 ml to a low of 66 ml. The headspace for the BF, Control, and DB and was statistically significantly different from the other bottles. The surface area of the milk-to-air interface was higher for AN, BF, EL, and PD than the other bottles and statistically showed significant differences. As the bottles were tipped to allow milk to flow out, the milk-to-air interface increased as an ellipse formed. As the milk was removed from the Control bottle via pipette, no bubbles formed on the milk-to-air-interface.

**Table 1:** Bottle measurements for diameter, headspace, and milk-to-air interface

**Delivery system**	**Diameter** ** (mm)**	**Headspace** ** (ml)**	**Milk-to-air interface** ** (cm^2^)**
Gerber (G)	52	176	21.23
Ventaire (V)	54	183	22.89
Dr. Brown's (DB)	50	66*	19.63
Elan (EL)	67	180	35.24*
BornFree (BF)	65	127*	33.17*
Playtex Drop-Ins (PD)	56	158	24.62*
Avent Natural (AN)	66	205	34.19*
Control	52	74*	20.42

*Statistically significant at p < 0.01

### Milk samples

The mean concentrations of ascorbic acid measured in the human milk samples, as seen in Table 2, are reported in milligrams per liter. The baseline values did not show statistically significant differences between groups. All milk samples had baseline mean concentrations of ascorbic acid between 41.87 and 42.66 mg/L. Mean concentrations of ascorbic acid in human milk from all bottles except Control, were significantly different from baseline to 20 minutes. At 20 minutes the bottle systems had mean concentrations of ascorbic acid between 31.83 and 0.49 mg/L, compared to 38.32 mg/L in the Control. This is a significant decrease in ascorbic acid concentration, with a range from 76% to 1% of baseline values, respectively. The Control varied by less than 10% from the baseline value maintaining 91% of the mean concentration of ascorbic acid at the 20 minute time point.

**Table 2:** Mean ascorbic acid concentration in human milk delivered by bottle in milligrams per liter of milk with standard deviation in (SD)

**Bottle system**	**0 minutes**	**5 minutes**	**10 minutes**	**15 minutes**	**20 minutes**
Gerber (G)	41.93 (0.05)	36.19 (0.18)	26.85* (2.17)	16.57* (3.32)	10.33* (3.07)
Ventaire (V)	41.95 (2.02)	39.06 (0.14)	32.90* (2.61)	29.22* (2.97)	22.87* (0.44)
Dr. Brown's (DB)	41.87 (0.83)	39.22 (1.08)	38.82 (1.09)	36.49* (4.21)	31.83* (3.06)
Elan (EL)	42.12 (3.81)	37.51 (1.76)	19.22* (2.06	8.62* (1.99)	0.49* (0.23)
BornFree (BF)	42.54 (4.83)	36.15 (1.00)	30.68* (2.01)	18.42* (3.53)	12.27* (2.92)
Playtex Drop-Ins (PD)	42.66 (2.67)	30.58 (4.60)	21.44* (1.87)	13.96* (3.39)	0.77* (0.30)
Avent Natural (AN)	42.44 (3.05)	34.59 (3.89)	24.71* (1.56)	18.52* (3.78)	3.90* (2.07)
Control	41.98 (0.05)	40.93 (0.66)	40.53 (0.58)	39.39 (1.58)	38.32 (2.27)

*Statistically significant at p < 0.01

**Figure 1:**
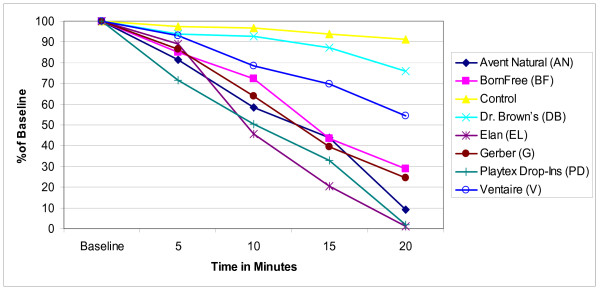
**Changes in mean ascorbic acid concentration in human milk**. Triplicate samples of human milk, standardized to 42 mg/L of ascorbic acid at baseline, were collected. The samples were analyzed for ascorbic acid using normal-phase high performance liquid chromatography and compared to a standard curve. The mean concentrations for each triplicate sample were calculated at baseline, 5, 10, 15, and 20 minutes. Results represent the mean values (± SD) of three experiments displayed as a percent of the baseline level.

**Figure 2:**
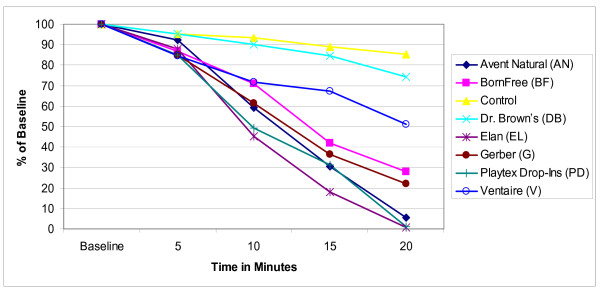
**Changes in mean ascorbic acid concentration in infant formula with iron**. Triplicate samples of infant formula with iron were collected. The samples were analyzed for ascorbic acid using normal-phase high performance liquid chromatography and compared to a standard curve. The mean concentrations for each triplicate sample were calculated at baseline, 5, 10, 15, and 20 minutes. Results represent the mean values (± SD) of three experiments displayed as a percent of the baseline level.

**Figure 3:**
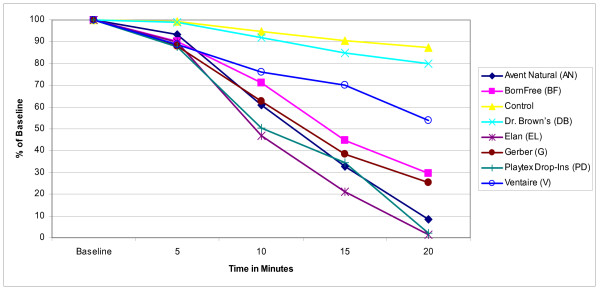
**Changes in mean ascorbic acid concentration in infant formula with docosahexaenoic acid**. Triplicate samples of infant formula with docosahexaenoic were collected. The samples were analyzed for ascorbic acid using normal-phase high performance liquid chromatography and compared to a standard curve. The mean concentrations for each triplicate sample were calculated at baseline, 5, 10, 15, and 20 minutes. Results represent the mean values (± SD) of three experiments displayed as a percent of the baseline level.

The mean concentration of ascorbic acid in human milk infant formula with iron, and infant formula with docosahexaenoic acid is reported in Figures 1, 2, and 3 as a percentage of the baseline level derived from the mean values of the triplicate samples for ease of comparison across and between groups. In all the bottle systems ascorbic acid did decline from baseline levels through the 20 minute samples, with a marked decline in three bottle systems (Figures 1, 2, and 3). For the human milk samples at five minutes, all ascorbic acid levels retained at least 82% of baseline levels. At the 10 minute time point the mean concentration of ascorbic acid decreased significantly below baseline for the AN, BF, EL, G, PD, and V bottles (Figure 1). At the 15 minute time point all bottles, except for the Control, had mean concentration decreases of up to 77% compared to baseline. Similar trends were seen for infant formula with iron and for infant formula with docosahexaenoic acid (Figures 2 and 3). At the 20 minute time point AN, BF, EL, G, and PD were all below 40% of baseline mean concentrations as seen in Figures 1, 2, and 3. The Control contained a mean ascorbic acid concentration of greater than 90% of the baseline concentration at the 20 minute sampling.

## Discussion

While the material for most of the bottles was polycarbonate, except for the BF and the PD, the bottles used in this study were quite different from each other in the way that air was replaced in the bottle as milk was removed. EL and V had removal screw-off bottoms with a diaphragm designed, according to the manufacturers, to prevent bubbles from forming in the milk. The BF bottle had a small venting system inside the collar of the nipple, which according to the manufacture, aids in preventing colic. Bubbles rapidly formed on the milk-to-air interface in the AN, BF, EL, G, PD, and V bottles as the milk was removed. No bubbles formed in the Control or the DB bottles.

The milk-to-air interface compared to the amount of headspace appeared to have a greater impact on the decrease of ascorbic acid levels than the amount of headspace. While Gliguem et al reported that the oxygen in the headspace of packaging was associated with degradation of ascorbic acid in stored milk [15], the headspace in the bottles did not correlate with the greatest decreases in mean ascorbic acid concentration. Those bottles with the largest milk-to-air interface did have the greatest decreases in mean concentration of ascorbic acid over time. The air moving through the milk and the formation of bubbles on the surface of the milk may be factors in the observed decreases of ascorbic acid concentration given that the Control had neither air moving through the milk nor bubbles forming on the surface. The Control bottle had the highest ascorbic acid concentration at 20 minutes.

## Conclusion

Infants require approximately 180 mL of human milk each day for each kilogram of body weight (180 mL/kg/day) [16]. The Food and Nutrition Board at the Institute of Medicine recommends an ascorbic acid intake for healthy infants aged 0 – 6 months of 40 mg/day and 50 mg/day for healthy infants age 6 months to 1 year [17], which in human milk equates to an estimated intake of ascorbic acid of 4 mg/100 mL of milk or approximately 7.2 mg/kg of body weight [18]. During the first 6 months of life most infants are able to meet this requirement by consuming human milk fed directly from the breast. Given the decreases in mean ascorbic acid concentrations observed in this study, an infant relying solely on either expressed human milk or infant formula that is delivered via a bottle there may be an increased risk of low intake of ascorbic acid. More research is needed to validate these findings as children with low intakes of ascorbic acid are more vulnerable to develop frequent and more severe common day-to-day infections [19].

Adults, as well as infants, rely on food for adequate ascorbic acid intake. It has been shown that adults who consume food containing ascorbic acid have increases in circulating concentrations of ascorbic acid within three hours. Sanchez-Moreno et al suggested that this increase in circulating ascorbic acid may play a role in the reduction seen in the formation of harmful compounds produced by oxidation reactions [20]. Heinonen et al reported that the plasma concentrations of ascorbic acid in a group of <32 week-gestation premature infants declined rapidly in the first three weeks of life [21]. These infants were fed pooled pasteurized human milk. In other studies, premature infants receiving formula had low plasma ascorbic acid concentrations [22,23]. Studies such as these underscore the importance of consistent intake of ascorbic acid, particularly in fragile populations such as premature infants being bottle-fed. The bottle systems used in this study had measurable decreases in the mean concentration of ascorbic acid over the 20 minute sampling period. More research is needed to determine if the observed decreases in ascorbic acid are related to plasma ascorbic acid concentration in infants exclusively bottle fed. Bottle shape, size, and venting (no air moving through the milk and no bubbles forming on the milk) should be considered when choosing a bottle system for infant feeding. Further research is needed regarding the handling and delivery of human milk with regard to preserving the integrity of specific nutrients.

## Abbreviations

AN: Avent^® ^Natural; BF: BornFree™; DB: Dr. Brown's Natural Flow^®; ^ EL: Evenflo^® ^Elan; G: Gerber^® ^Classic; PD: Playtex^® ^Drop-Ins; V: Playtex Ventaire^®.^

## Competing interests

Dr. Jimi Francis has served as a resource regarding breastfeeding issues for Handi-craft Company.

The other authors declare that they have no competing interests.

The authors declare that none of them own stock or shares in any organization that could benefit from this study. Nor do any of the authors hold or are applying for any patents relating to the content of this manuscript.

## Authors' contributions

KR carried out the extractions, participated in the sample processing and helped draft the manuscript. PB participated in the HPLC. DD participated in the sample collection and processing, input data, helped with statistical analysis, and helped draft the manuscript. JF conceived of the study, performed the statistical analysis, participated in the study implementation and helped to draft the manuscript. RP participated in the study design and coordination and helped to draft the manuscript. All authors read and approved the final manuscript.
